# Identification of a novel nidovirus as a potential cause of large scale mortalities in the endangered Bellinger River snapping turtle (*Myuchelys georgesi*)

**DOI:** 10.1371/journal.pone.0205209

**Published:** 2018-10-24

**Authors:** Jing Zhang, Deborah S. Finlaison, Melinda J. Frost, Sarah Gestier, Xingnian Gu, Jane Hall, Cheryl Jenkins, Kate Parrish, Andrew J. Read, Mukesh Srivastava, Karrie Rose, Peter D. Kirkland

**Affiliations:** 1 Virology Laboratory, Elizabeth Macarthur Agriculture Institute, Menangle, New South Wales, Australia; 2 Australian Registry of Wildlife Health, Taronga Conservation Society Australia, Bradleys Head Road, Mosman, New South Wales, Australia; 3 Microbiology and Parasitology, Elizabeth Macarthur Agriculture Institute, Menangle, New South Wales, Australia; Consejo Superior de Investigaciones Cientificas, SPAIN

## Abstract

In mid-February 2015, a large number of deaths were observed in the sole extant population of an endangered species of freshwater snapping turtle, *Myuchelys georgesi*, in a coastal river in New South Wales, Australia. Mortalities continued for approximately 7 weeks and affected mostly adult animals. More than 400 dead or dying animals were observed and population surveys conducted after the outbreak had ceased indicated that only a very small proportion of the population had survived, severely threatening the viability of the wild population. At necropsy, animals were in poor body condition, had bilateral swollen eyelids and some animals had tan foci on the skin of the ventral thighs. Histological examination revealed peri-orbital, splenic and nephric inflammation and necrosis. A virus was isolated in cell culture from a range of tissues. Nucleic acid sequencing of the virus isolate has identified the entire genome and indicates that this is a novel nidovirus that has a low level of nucleotide similarity to recognised nidoviruses. Its closest relatives are nidoviruses that have recently been described in pythons and lizards, usually in association with respiratory disease. In contrast, in the affected turtles, the most significant pathological changes were in the kidneys. Real time PCR assays developed to detect this virus demonstrated very high virus loads in affected tissues. *In situ* hybridisation studies confirmed the presence of viral nucleic acid in tissues in association with pathological changes. Collectively these data suggest that this virus is the likely cause of the mortalities that now threaten the survival of this species. Bellinger River Virus is the name proposed for this new virus.

## Introduction

The Bellinger River snapping turtle, *Myuchelys georgesi*, is a species of freshwater turtle that, prior to this outbreak, was rare and has a very restricted habitat. It is confined solely to a 60 kilometre section of the Bellinger River, and a short section of the adjacent Kalang River in northern coastal New South Wales (NSW), Australia. While the turtle had been described as “locally abundant” it was also described as “meets the criteria for being listed as a vulnerable species under the Threatened Species Conservation Act of NSW” [1]. In 2014 it was estimated that there were approximately 2500 of this species in the wild [2]. Commencing in mid-February 2015, a number of people using and managing the environment surrounding the Bellinger River observed a large number of deaths in *M*. *georgesi*. A multi-agency investigation was undertaken to establish the cause and extent of the outbreak. Mortalities continued for 7 weeks and involved mostly adult animals. More than 400 dead or dying animals were observed and population surveys conducted after the outbreak had ceased indicated that only a small proportion of the total population had survived with very few adults remaining. No other species, including the sympatric Murray River turtle (*Emydura macquarii*), appeared to be affected. Full details of the prevailing environmental conditions and the extent of the investigation have been described by others [2]. A number of moribund and dead *M*. *georgesi* were collected for post mortem examination at the Australian Registry of Wildlife Health, Taronga Conservation Society Australia Mosman, NSW. Tissue samples were referred to a number of different laboratories to test for a wide range of potential pathogens, toxins and water quality assessment. No consistent results were obtained from bacterial cultures and tests for mycoplasma, trichomonas, chlamydia and toxins gave negative results. On the basis of the histological changes and a failure to identify any other causative agent, a viral aetiology was suspected. Testing was undertaken at several other laboratories and ranaviruses, adenoviruses, paramyxoviruses (ferlavirus) and herpesviruses were excluded [2]. Samples were also referred to the Elizabeth Macarthur Agriculture Institute, Menangle NSW. Here we report the isolation and characterisation of a novel nidovirus and provide evidence for its involvement as the principal pathogen in this disease outbreak.

## Materials and methods

### Animal ethics approval

The NSW Office of the Environment and Heritage (OEH) was consulted during the early stages of the investigation and confirmed that, as this was a diagnostic investigation, no Animal Care and Ethics (AEC) or other approvals were necessary. When sample collection was undertaken as part of the follow-up epidemiology investigations, all procedures were approved by the AEC of the Taronga Conservation Society of Australia (AEC approval number 3e/10/15).

### Specimen collection

Twenty one turtles that were either dead or moribund and then euthanased were submitted to the Australian Registry of Wildlife Health, Taronga Conservation Society of Australia where post mortem examinations were conducted. Various samples from each of the 21 *M*. *georgesi* were submitted to the Virology Laboratory at the Elizabeth Macarthur Agriculture Institute. Swabs had been collected from the buccal cavity (n = 5), conjunctiva (n = 5) and cloaca (n = 5) and placed in 3mL of phosphate buffered gelatin saline (PBGS, pH 7.3) and held at 4°C. Samples of brain (n = 4), conjunctiva (n = 5), kidney (n = 10), liver (n = 10), lung (n = 9), myocardium (n = 5), ovary (n = 2), spleen (n = 10), urinary bladder (n = 5) and serum or plasma (n = 11) (either fresh or after holding frozen at -80°C) were sent on frozen blocks for ‘same day’ delivery to the Elizabeth Macarthur Agriculture Institute. Samples of frozen kidney (n = 5) or liver (n = 3) were also submitted from an archival collection of *M*. *georgesi* tissues that had been held at The Australian Museum, Sydney since collection in 1991. Suspensions (approximately 20%, w/v) of fresh tissues from affected animals were prepared by homogenising in serum free Minimal Essential Medium (MEM), containing penicillin (230μg/mL), streptomycin (250μg/mL) and amphotericin-B (5μg/mL), using a bead beater. The supernatant was collected after centrifugation at approximately 3000g for 20 min at 4°C and filtered using a 0.45μm syringe filter. A wide range of tissues was also collected and fixed for 48 hours in 10% neutral buffered formalin solution.

### Histopathology

Sections of formalin fixed tissue were cut and stained with haematoxylin and eosin, Giemsa and Periodic acid-Schiff stains using standard methods and examined by light microscopy.

### Virus isolation

Sub-confluent monolayer cultures of Buffalo African green monkey kidney (BGM) cells [3] initially grown at 37°C in 10mL cell culture tubes containing MEM supplemented with 10% (v/v) foetal bovine serum (FBS), penicillin (115μ/mL), streptomycin (125μg/mL) and amphotericin-B (2μg/mL) were used for the first virus isolation attempts. Immediately before use the culture medium was replaced with fresh maintenance medium (MEM, 2% FBS and antibiotics) and 200μL of filtered supernatant from tissue homogenate was added to the medium. Based on experience with other aquatic pathogens, all cultures were maintained at 25°C. Cells were observed frequently and passaged after 7 days by scraping the cells off the tube surface and adding 200μL of suspension to new sub-confluent monolayers. After changes were observed in the morphology of BGM cell monolayers, tissue culture fluids were also passaged onto sub-confluent monolayers of other mammalian, avian, fish, reptile or mosquito cell cultures. Full details of the cell cultures used and the relevant culture conditions are summarised in Table 1.

**Table 1:** Cell cultures used for virus isolation and susceptibility studies with culture conditions that were employed.

Order	Species/ organ	Cell culture	Defined medium	Growth temp	Maint. temp	Comments	Reference
Mammalian	Monkey kidney	BGM	MEM	37°C	25°C		[3]
	Monkey kidney	CV-1	RPMI 1640	37°C	25°C		ATCC* CCL-70;
	Monkey kidney	Vero	Medium 199	37°C	25°C		ATCC CCL-81
	Bovine kidney	MDBK	MEM	37°C	25°C		ATCC CCL-22
	Hamster Lung	HmLu-1	MEM	37°C	25°C		[4]
Avian	Chicken embryo fibroblast	CEF	MEM	37°C	25°C	Primary cell culture	NA
Fish	Fat head minnow	FHM	MEM	25°C	25°C		ATCC CCL-42
	Sea bass	SB	MEM	25°C	25°C		[5]
	Striped snakehead	SSN-1	Liebovitz L15	25°C	25°C	Plus extra glutamine	ECACC** 96082808
Insect	Mosquito	*Aedes albopictus*—C6/36	Medium 199	30°C	25°C		ATCC CRL-1660
Reptile	Viper heart	VH2	MEM	30°C	25°C	Plus non- essential amino acids	ATCC CCL-140

* American Type Culture Collection

**European Collection of Authenticated Cell Cultures

The defined medium for all cell cultures was supplemented with 10% foetal bovine serum and antibiotics (penicillin 115μg/mL, streptomycin 125μg/mL, amphotericin B 2μg/mL). The serum concentration was reduced to 2% for maintenance.

### Electron microscopy

Cell culture supernatants were examined by electron microscopy by placing 10μL of sample on parlodion/carbon coated 400 mesh copper grids. After washing briefly with de-ionised water, negative staining was achieved by the addition of 2% aqueous uranyl acetate. The stained specimens were examined in a Philips 208 transmission electron microscope.

### Nucleic acid sequencing

Culture supernatant from BGM cell cultures showing advanced cytopathology was clarified by centrifugation at 3,000g for 30 min and passed through a 0.45μm filter. The virus was then pelleted at 25,000g for 2 hours at 4°C and resuspended in 500μL of nuclease free water. Host cell nucleic acids were removed by incubating with DNase I (250U; Stratagene) and an RNase Cocktail (0.5U RNase A and 20U RNase T1; Ambion) at 37°C for 2 hours. Total ribonucleic acids were then extracted and eluted in 20μL of RNase free water using an RNeasy minikit (Qiagen). A DNA library was prepared using a TruSeq Stranded mRNA Sample Prep Kit, omitting the poly (A) mRNA purification step and sequencing with 150bp paired end reads was completed on an Illumina Miseq platform at the Australian Genome Research Facility (Brisbane, Australia). Raw NGS data has been submitted to the Sequence Read Archive (SRA) (Accession SRP158959) [6].Trimmomatic software [7] was used to determine and remove low quality bases and adapter sequences using a minimum quality score of 20. Scaffolds (nodes) were then assembled in Velvet Optimiser [8] and run in the Megablast 2.2.26 software [9] to identify homology with sequences in the Genbank database [10]. Some scaffolds were identified as having homology with ball python nidovirus sequences. To remove extraneous sequence data only the trimmed sequences that aligned to ball python nidovirus genome (KJ541759), using Burrows-Wheeler Aligner [10], were assembled into scaffolds using VelvetOptimiser [8]. The scaffolds were then aligned in Sequencher [11] to form a single contig. Additional Sanger sequencing was completed after further amplification using the primers listed in S1 Table.

### Phylogenetic analysis

Open reading frames (ORFs) were determined from the full sequence using Open Reading Frame Viewer software [12]. Similarity to other viruses for each of the ORFs and their predicted amino acid sequences were determined by searches using BLASTn and BLASTp [13] algorithms through the NCBI server (http://blast.ncbi.nlm.nih.gov/Blast.cgi).

A region of the ORF1b which was predicted to code for an RNA-dependant RNA polymerase (RdRp) was selected for comparative studies because this region was found to be the most highly conserved across the *Torovirinae* subfamily. The amino acids of this conserved region of the ORF1b, were aligned against other members of the *Torovirinae* and representatives from the other families within the order *Nidovirales*.

A phylogenetic tree was produced using the Maximum Likelihood method based on the JTT matrix-based model [14] with 1000 bootstrap replicates. Initial trees for the heuristic search were obtained automatically by applying the Neighbor-Joining method to a matrix of pairwise distances estimated using a JTT model. The analysis involved amino acid sequences from 44 virus strains. All positions containing gaps and missing data were excluded. There was a total of 268 amino acid positions in the final dataset. Phylogenetic analyses and percentage similarities were calculated using Clustal W alignment in MEGA6 [15].

Using the methods described above, similar comparisons were made for the conserved putative helicase (342 positions) and M^pro^ C-like (302 positions) domains of polyprotein 1ab, as well as the whole length polyprotein 1a and spike protein.

### Real time PCR assay

After the detection of a novel RNA viral sequence by NGS, qRT-PCR Taqman assays were designed to detect nucleic acid sequences that were specific for the novel virus and directed at the sequence encoding the presumptive polyprotein 1a (replicase 1a). The resulting assay was used to test serum and a range of tissue homogenates from affected animals. Later, a selection of samples was also tested in an assay directed at the region encoding the ‘spike’ protein. For testing of samples in these assays, total nucleic acid was extracted using a magnetic bead based kit and a magnetic particle handling system [16]. The details of primers and probe are as follows:

### Polyprotein 1a (replicase 1a) assay

Forward Primer—TNID F: (2536–2555) 5’GGGAGCGACTGATCTGTTTG3’

Reverse Primer—TNID R: (2478–2497) 5’TCACCGCAGGTATCACAATC3’

Probe—TNID Pr: (2506–2533) FAM 5’CAGTGTACATGTTTCGGATGGTTTGAGT3’ BHQ1

### Spike protein assay

Forward—TNV spike F: (25841–25861) 5’GCAAGCCTCAACAGCATCATC3’

Reverse–TNV spike R: (25899–25918) 5’GCCGCAGACTAGGAACCATT3’

Probe—TNV spike Pr: (25863–25885) Quasar670 5’CCCAAATCAACGCCTGGTCGATC3’ BHQ2

The qRT-PCR assays, both of which produced a 77bp product, used AgPath Mastermix (Life Technologies) and was run on an ABI 7500 thermocycler for 45 cycles under standard conditions as specified by the mastermix manufacturer. On each occasion the assay was run, included on the plate were two positive control samples, representing approximately 10000 (PC1) and 1000 (PC2) copies of viral RNA, a negative control (NC, a tRNA solution) and a ‘no template’ control (NTC), the latter consisting of nuclease free water. The positive and negative controls were included throughout the procedure from extraction through to the completion of the PCR reactions while the NTC was only added during the PCR setup. An exogenous RNA control [16] was also included to monitor the efficiency of the nucleic acid extraction and PCR reaction and to detect the presence of inhibitors. Any evidence of RNA amplification was recorded and results were expressed as cycle threshold (Ct) values as described previously [16].

### *In situ* hybridisation

A conventional RT-PCR was run to produce template for *in situ* hybridisation (ISH) probe production. PCR template was created using conventional PCR primers targeting the gene encoding a putative viral membrane protein (M). Primer details were as follows: BRVMP forward: 5’ ATGGAGTCCACCTCGA 3’, BRVMP reverse: 5’ TTATGGTAGGATGGCTGTT 3’.

RT-PCR reactions were prepared using the MyTaq One-Step RT-PCR Kit (Bioline) according to the manufacturer’s instructions and PfuUltra II fusion DNA polymerase (Agilent) was added to the reaction at a final dilution of 1/100. The template used (5μL) was purified from the cell culture isolate as described previously. Cycling parameters for the PCR assay were a 20 min initial reverse transcription step at 45°C and a 1 min denaturation step at 95°C, followed by 40 cycles of denaturation at 95°C for 10s, annealing at 56°C for 30s, extension at 72°C for 30s and a final extension step at 72°C for 7 min. The resulting template (of approximately 650 base pairs) was visualised using electrophoresis in a 1.5% agarose gel stained with Gel Red Nucleic Acid Gel Stain (Biotium).

Approximately 1ng of PCR product was used in the PCR digoxigenin (DIG) ISH Probe Synthesis Kit (Roche) with thermocycling parameters as described in the kit instructions. The size of the DIG-labelled probe was determined using electrophoresis (as described above) and was quantified using a Nanodrop spectrophotometer (Thermo Fisher).

A Hybaid Omnislide thermal cycling system (Thermo Scientific) was used to maintain humidity and temperature control during all incubation steps. Hybrislip coverslips (Sigma) were used at every step. Sections of tissue 4μm thick were cut onto Superfrost Plus slides (Menzel Gläser) and dried at 65°C for 30 min. Slides were dewaxed in xylene and rehydrated in an ethanol series. Slides were treated with 100 μL Proteinase K (Dako) for 15 min at 37°C, followed by a 3 min Tris buffer wash (0.1 M, pH 8.0) and pre-hybridisation for 1h at 37°C in 100 μL prehybridisation buffer (50% formamide, 1 x Denhardt’s solution, 4 x SSC, 0.25 mg yeast tRNA/mL, 10% dextrans sulfate). An exchange was made with 100 μL hybridization buffer containing prehybridisation solution with 5 ng/μL ISH probe and slides were heated at 95°C for 5 min. Slides were cooled on ice for 5 min then incubated overnight at 42°C. The next day slides were washed in wash buffer (0.1 M maleic acid, 0.15 M NaCl and 0.3% Tween20, pH 7.5) (Roche) at 40°C for 10 min. Sections were then blocked with 500 μL blocking buffer (1% Roche blocking reagent in wash buffer) at room temperature (RT) for 30 min. Blocking buffer was exchanged with a 1/200 dilution of anti-DIG antibody (Roche) in blocking buffer and incubated for 1 hr. Sections were washed for 30 min at RT in wash buffer and equilibrated for 5 min at RT in detection buffer (Roche) and incubated with 500 μL BCIP/NBT in the dark at RT for 5 h. Slides were then rinsed in tap water and counterstained with 0.2% Fast Green (Australian Biostain) for 20 sec and mounted in DPX mounting medium (Sigma-Aldrich). Negative control slides included additional tissue sections processed without the ISH probe and sections with ISH probe applied that were taken from an Eastern long neck turtle (*Chelodina longicollis*) that had given negative results by PCR. The latter samples were included in the absence of fixed tissues from presumptively uninfected *M*. *georgesi*.

### Field survey

Following the detection of the novel virus, in November 2015 (about 6 months after the cessation of the outbreak) an intensive survey of the parts of the river where affected turtles had been detected [2] was undertaken by groups of biologists and ecologists and samples collected from a wide range of aquatic species and some terrestrial animals (n = 360) to establish the size of the remaining population and whether any other animals were carrying this virus. A total of 502 samples were collected, consisting of various amphibians, arthropods, fish and reptiles. For animals of sufficient size, swabs were collected separately from mucosal surfaces (usually conjunctiva, oral and cloaca), placed in PBGS and held at approximately 4°C for up to 10 days before being sent to the laboratory. Blood samples were also collected from larger animals (n = 83) and serum was submitted for testing. Small invertebrates and small vertebrates were preserved in absolute ethanol. Pooled tissues or whole bodies from ethanol fixed animals were prepared for nucleic acid extraction by first digesting in proteinase K solution as described previously [17]. Full details of the species, the samples collected and the numbers examined are listed in Table 2.

**Table 2:** Species sampled from the Bellingen River November 2015 field survey.

Class	Scientific Name (Common Name)	Animals Tested	Positive by qRT-PCR* (%, 95% CI)	Cycle Threshold* (Ct) (Ave, range)
Reptilia	*Myuchelys georgesi* (Bellinger River snapping turtle)	31	9 (29; 14–48)	Av: 35.42 (30.39–38.36)
	*Emydura macquarii* (Murray River turtle)	49	2	36.95,37.34
	*Intellagama lesueurii* (Eastern water dragon)	5	0	
	*Lampropholis guichenoti* (garden skink)	1	0	
	*Dendrelaphis punctulatus* (green tree snake)	1	0	
Actinopterygii	*Gobiomorphus coxii* (Cox’s gudgeon)	7	0	
	*Notesthes robusta* (bullrout)	1	0	
	*Pseudomugil signifier* (Southern blue-eye)	49	0	
	*Gambusia holbrooki* (Eastern gambusia)	27	0	
	*Melanotaenia duboulayi* (crimson-spotted rainbowfish)	1	0	
	*Retropinna semoni* (Australian smelt)	1	0	
	*Anguilla reinhardtii* (long finned eel)	5	0	
Amphibia	*Mixophyes fasciolatus* (adult great barred frog)	1	0	
	*Litoria caerulea* (green tree frog)	1	0	
	*Litoria wilcoxii* (Wilcox’s frog)	55	0	
	*Adelotus brevis* (adult tusked frog)	1	0	
	*Mixophyes sp*. (tadpoles–barred frogs)	13	0	
	Unknown species (tadpoles)	26	0	
Malacostraca	*Macrobrachium australiense (*freshwater prawn)	3	0	
	Australatya striolata (rifle shrimp)	1	0	
	Cherax destructor (yabbie)	1	0	
	Unknown species (shrimp)	20	0	
Insecta	Trichoptera (caddisfly larvae)	3	0	
	Eggs/casings (unknown species)	13	2	37.18, 38.36
Bivalvia	Unknown species (mussels)	8	0	
Gastropoda	Unknown species (snails)	2	0	
Clitellata	Unknown species (Hirudinea–leech)	34	0	
**Total**		**360**	**13**	

* Refer to Methods section for details of the qRT-PCR assay and interpretation of Ct values.

## Results

### Pathology findings

When examined at necropsy, animals were usually in poor body condition, had bilateral swollen eyelids and conjunctivitis. Some animals had tan foci on the skin of the ventral thighs. Consistent histological findings included severe peri-orbital, splenic and nephric inflammation and necrosis. A proportion of affected animals also had evidence of a fibrinoid vasculopathy. Full details of the gross and histopathology will be described elsewhere.

### Virus isolation

**Fig 1:**
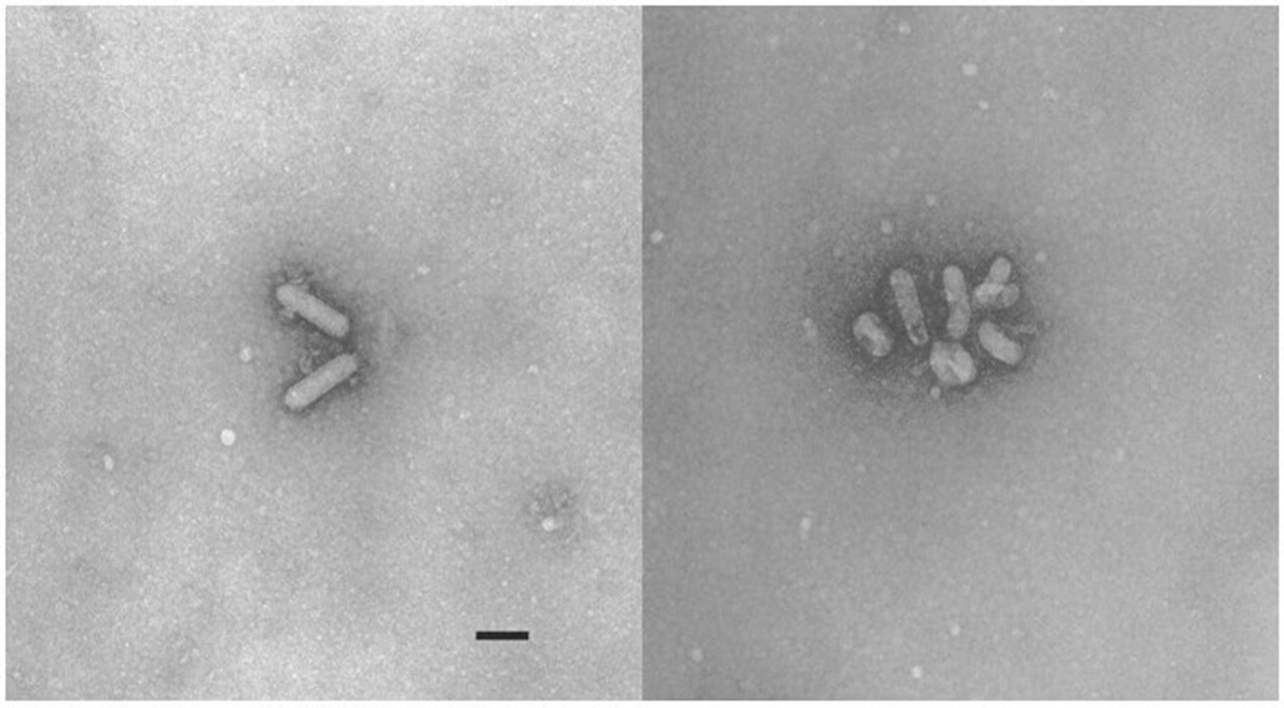
Electron micrographs of Bellinger River nidovirus particles in culture supernatant of infected CV-1 cells. (Scale bar = 100 nm).

When BGM cells were inoculated with pooled homogenates of spleen and lung tissues from 5 animals, after 2 passages cytopathology consisting of lytic destruction of cells in small foci was observed after approximately 5 days. Following further passage, more widespread evidence of cytopathology was detected, affecting approximately 50% of the monolayer after 4–5 days. Passage of supernatant from infected BGM cultures onto CV-1 cell monolayers resulted in extensive destruction of the monolayer within 48–72 hours. Limited cytopathology was observed in Vero cells while no microscopic evidence of virus replication was detected in hamster lung (HmLu-1), avian (CEF), fish (SB, FHM, SSN-1), reptile (VH2) or mosquito (C6/36) cell lines. In subsequent studies, this virus replicated in MDBK cells but without cytopathology. Replication could only be detected by PCR and virus loads were lower than those obtained in CV-1 cells. Examination of the culture supernatant from CV-1 cells by electron microscopy identified bacilliform particles approximately 170 nm long and 25 nm wide (Fig 1). The name “Bellinger River virus” (BRV) is proposed for this virus.

Subsequent to the successful detection of virus in the pool of tissues, further virus isolation attempts were undertaken on a number of individual tissue samples. Viruses were isolated in cell culture from heart (10/10 samples), kidney (11/14), plasma (2/4) and spleen (4/5).

### Nucleic acid sequencing

**Fig 2:**
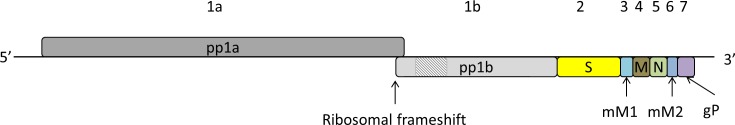
Genome organisation of Bellinger River virus. Diagrammatic representation of Bellinger River virus genome organisation and expression based on a comparison with the genome organisation for other viruses in the proposed *barnivirus* genus. ORFs are represented by boxes with the number above. The putative encoded protein acronyms are included within the boxes. pp1a, polyprotein 1a; pp1b, polyprotein 1b; S, spike protein; mM1, minor membrane protein 1; M, membrane protein; N, nucleoprotein; mM2, minor membrane protein 2; gP, glycoprotein; Hel, helicase; RdRp, RNA-dependent RNA polymerase and M^pro^, main proteinase.

The nucleic acid sequencing analysis identified a sequence of 30742 nucleotides of a virus with a genome organisation most closely related to viruses from the family *Coronaviridae* and subfamily *Torovirinae* (Fig 2). This sequence was subsequently confirmed by primer walking and further Sanger sequencing to establish what is considered to be the full length sequence (Genbank reference MF685025) of a nidovirus. The pattern of ORFs of this virus closely matches the pattern observed for other nidoviruses. However, this appears to be a novel nidovirus with less than 50% nucleotide similarity to recognised nidoviruses.

**Fig 3:**
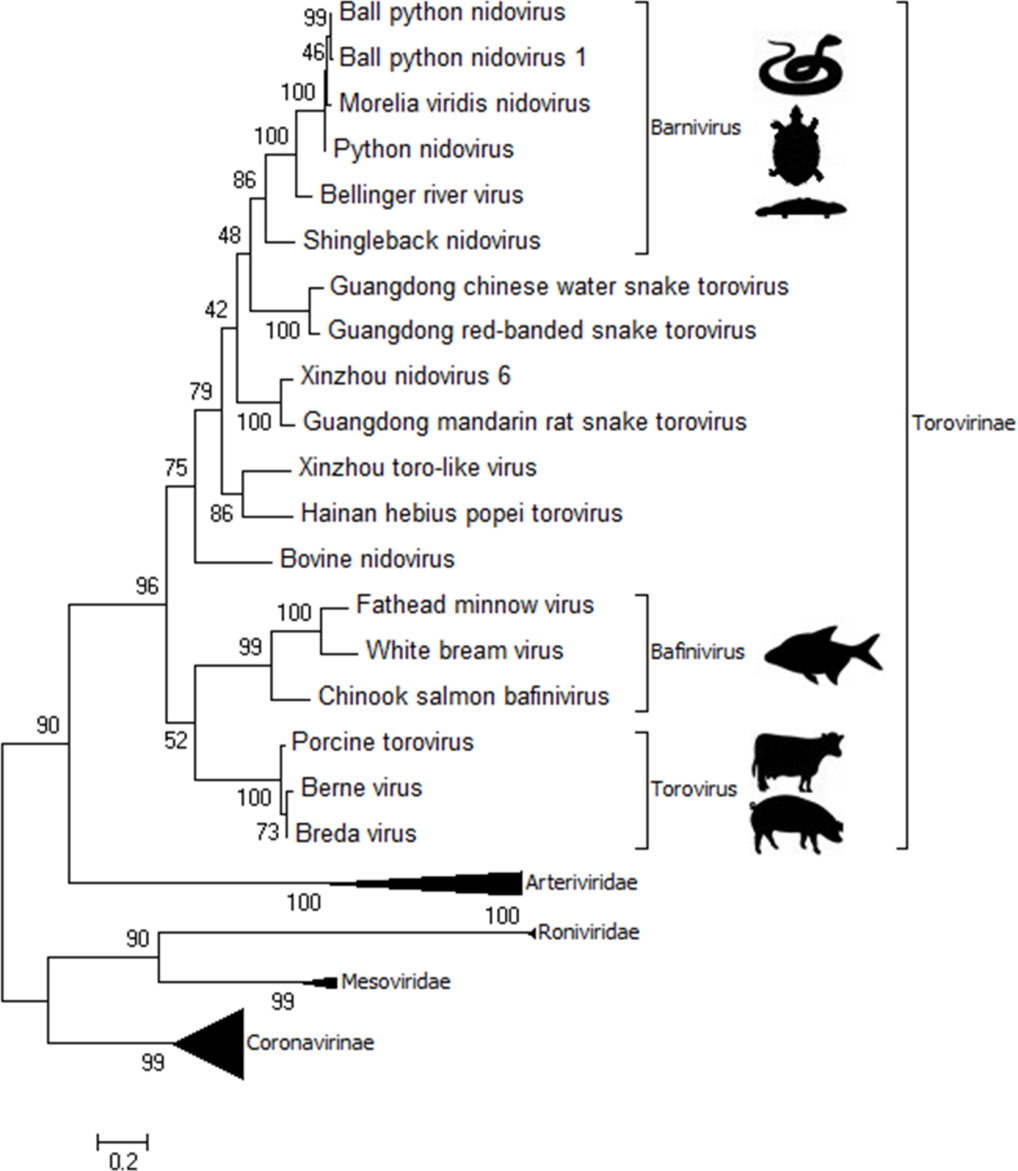
Phylogenetic tree showing the genetic relationships of members of the sub-family *Torovirinae* based on the conserved RdRp site of polyprotein 1b. Representative strains of the order Nidovirales are also included. The comparison was based on the alignment of 268 amino acid positions within the conserved RNA-dependent RNA polymerase (RdRp) region of polyprotein 1b. Phylogenetic tree was inferred with 1000 bootstrap replicates using the Maximum Likelihood method based on the JTT matrix-based model [14]. Branch lengths are in scale to the scale bar. The percentage bootstrap value associated with each lineage is indicated. The viruses included were ball python nidovirus [AIJ50565.1]; ball python nidovirus 1 [KM267236]; Morelia viridis nidovirus [ASO76150.1]; python nidovirus [AII00825.1]; Bellinger river virus [MF685025]; Shingleback nidovirus [AOZ57153.1]; Xinzhou nematode virus 6 [APG77345.1]; Xinzhou toro-like virus [YP 009344970.1]; Hainan hebius popei torovirus [MG600028]; Guangdong chinese water snake torovirus [MG600029]; Guangdong red-banded snake torovirus [MG600030]; Guangdong mandarin rat snake torovirus [MG600031]; Bovine nidovirus [YP 009142787]; Fathead minnow nidovirus [ADN95978.2]; White bream virus [YP 803213]; Chinook salmon bafinivirus [AVM87336.1]; Porcine torovirus [NC 022787.1]; Berne virus [P0C6V7.1]; Breda virus [YP 337905.2]; members of Arteriviridae, Mesoniviridae, Roniviridae, Coronaviridae are provided in S2 Table, along with aligned amino acid residues.

Phylogenetic analysis using aligned amino acid sequences from the conserved RdRp site of polyprotein 1b (pp1b) confirmed the classification of the virus and places it in the proposed *barnivirus* (bacilliform reptile nidovirus) genus [18] (Fig 3). Very similar results were obtained from comparisons involving the conserved putative helicase and M pro C-like domains of polyprotein 1ab, as well as the whole length polyprotein 1a and spike protein (S1–S4 Figs).

This new nidovirus appears most closely related to ball python nidovirus 1 [18, 19], python nidovirus [20] and *Morelia viridus* nidovirus [21], however these viruses have yet to be assigned to a genus. The highest amino acid sequence similarity was found in the pp1b region (68–69%), while all other proteins had lower levels of similarity (Table 3).

**Table 3:** Percentage amino acid identity between BRV and members of the proposed *barnivirus* genus.

	Coding regions for *barnivirus* genes							
Virus	pp1a	pp1b	Spike	mM1	M	N	mM2	HN
**BPN**	**53%**	**68%**	**45%**	**35%**	**53%**	**41%**	**26%**	**41%**
	79%	99%	99%	54%	93%	86%	75%	87%
**PN**	**53%**	**69%**	**45%**	**25%**	**51%**	**43%**	**26%**	**38%**
	79%	99%	99%	91%	29%	86%	92%	95%
**MVN**	**54%**	**69%**	**45%**	**27%**	**52%**	**40%**	**28%**	**38%**
	79%	99%	99%	85%	94%	94%	77%	96%
**SBN**	**33%**	**48%**	**30%**	**N/A**	**37%**	**N/A**	**23%**	**N/A**
	60%	99%	85%	N/A	93%	N/A	95%	N/A

Alignment and similarity modelling performed using BLASTp [13], using BLOSUM62 Matrix; gap cost for existence = 11, extension = 1; expected threshold = 10; word size = 3. Upper number (bold) indicates the percentage amino acid identity, while the bracketed lower number indicates the percentage coverage of the putative protein.

BPN–Ball python nidovirus [MG752895, KJ541759]

PN—Python nidovirus [KJ935003]

MN—Morelia viridis nidovirus [NC_035465]

SBN–Shingleback nidovirus [KX184715]

BLAST search also identified lower levels of homology with shingleback nidovirus (SBNV)[22], a virus of Australian shingleback lizards, two virus sequences that were identified from intestinal nematodes in Asian snake species [23], and four virus sequences identified in Asian snake species [24]. These viruses are likely to also be classified within the proposed genus *barnivirus*.

**Fig 4:**
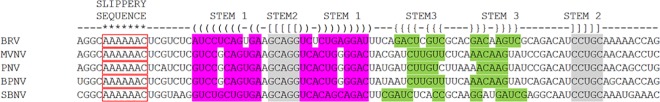
Sequence alignments for the 5 members of the proposed genus *barnivirus* that have been associated with disease. The conserved presence of a slippery heptanucleotide sequence (AAA AAA C), is indicated with an *. Base pairings in stem 1 are indicated with ‘()’s and magenta background, base pairings in stem 2 of the pseudoknot are indicated with ‘[]’s and grey background, while base pairings in stem 3 are indicated with ‘{}’s. BRV, Bellinger River virus; MVNV, Morelia viridis nidovirus; PNV, python nidovirus; BPNV, ball python nidovirus;.

In common with most other members of the *Coronaviridae* [25–27] a ribosomal frameshift sequence was identified within the first ORF of the turtle nidovirus sequence. An alignment of this region across the possible members of the proposed *barnivirus* genus is shown in Fig 4.

**Fig 5:**
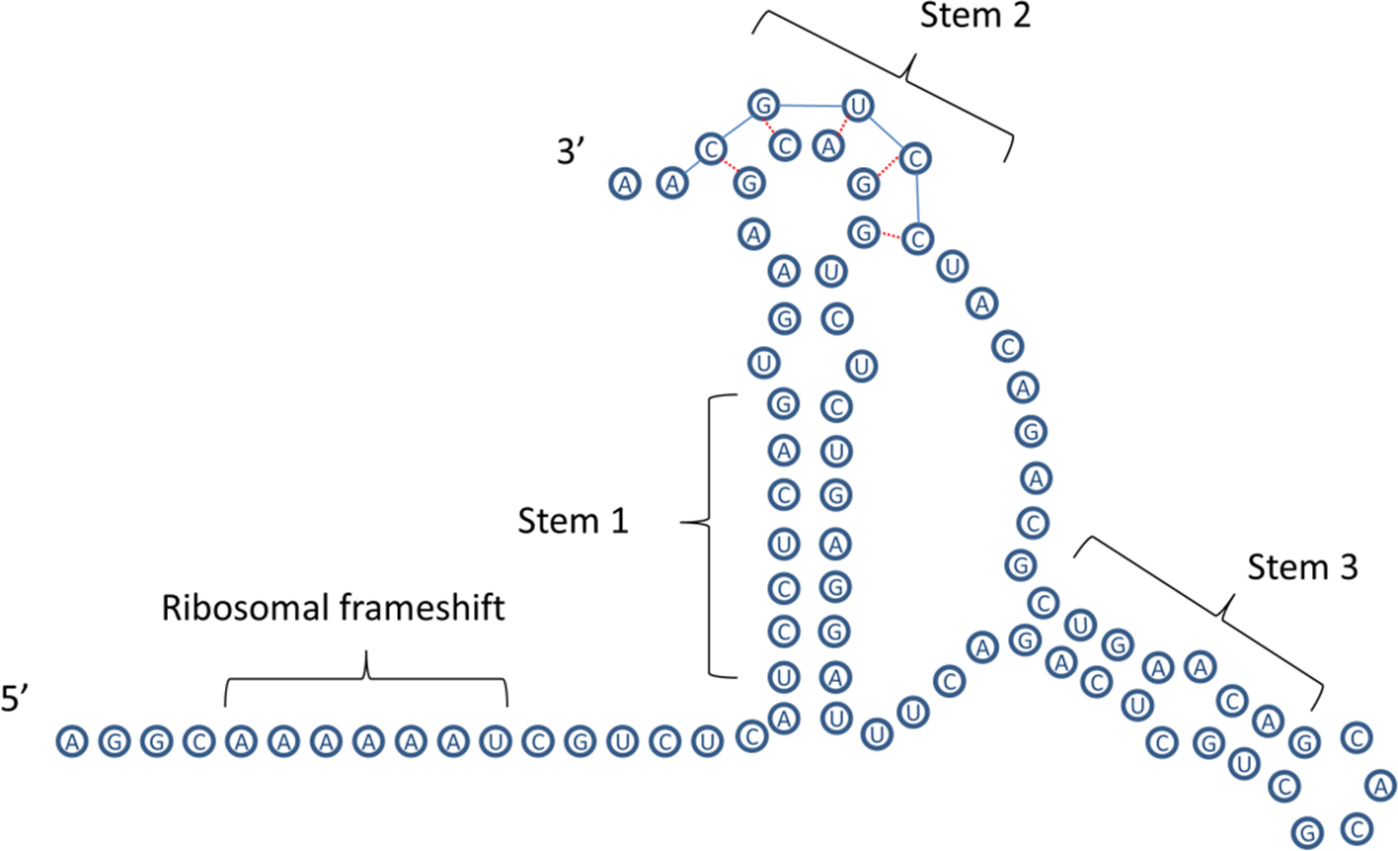
Predicted RNA secondary structures for the ribosomal frameshift sequence of Bellinger river virus. The “kissing stem loop” [28] is indicated by Stem 2.

Watson-Crick base pairing of the RNA sequence shows a common pseudo-knot motif downstream to the ribosomal frameshift sequence. This pseudoknot secondary structure is postulated to play an important role in the discontinuous synthesis of subgenomic RNAs [28]. The structure of this pseudo-knot differs from most other similar structures in coronaviruses by the presence of an additional stem in the region between the first stem and the “kissing stem loop”. This predicted structure is common to all of the putative members of the proposed *barnivirus* genus [18, 29] (Fig 5).

The qRT-PCR assay that was developed to detect the presumptive replicase 1a protein coding region of this virus allowed the detection of viral RNA in tissues from affected animals. Samples of kidney, liver, lung and spleen from the 5 animals that had contributed to the virus isolation pool were tested. Very high virus loads were detected in kidneys and spleen (Ct range 15.9–16.4) of 2 of the 5 animals but variable and moderately high levels of viral RNA were also detected in other tissues of these and the other 3 animals (Table 4, animals 1–5). Testing of a wider range of tissues from another 5 dead animals from the outbreak demonstrated high virus loads in heart, kidney, lung and spleen while virus was present in all other tissues at moderate levels (Table 4, animals 6–10). Relatively high levels of viral RNA were also detected in oral, conjunctival and cloacal swabs of these animals (Table 5). Serum samples from another 11 animals gave strong to moderate reactivity (Ct range 19.4–35.6) (Table 6). Very similar results were obtained for all samples when tested in the spike protein assay. As the reactivity in both assays was extremely similar, the extracts of the archival samples were only tested in the replicase 1a qRT-PCR and gave negative results.

**Table 4:** qRT-PCR results for tissue homogenates collected from affected *M*. *georgesi* turtles using an assay detecting a segment of the putative replicase 1a gene. Results for samples with very high RNA loads (Ct<20) shown in bold.

Animal	Kidney	Liver	Lung	Spleen	Bladder	Brain	Eye	Heart	Ovary
1	**15.92**	28.56	ND	25.08	NT	NT	NT	NT	NT
2	**15.97**	21.28	23.32	**16.40**	NT	NT	NT	NT	NT
3	27.92	33.50	35.64	30.01	NT	NT	NT	NT	NT
4	20.70	32.79	38.34	27.58	NT	NT	NT	NT	NT
5	21.51	23.78	27.42	21.84	NT	NT	NT	NT	NT
6	27.40	23.46	28.70	24.13	33.94	20.88	**19.49**	25.76	23.77
7	26.53	27.65	23.99	22.41	29.34	21.80	20.84	28.26	21.85
8	**14.89**	20.24	**17.25**	**14.96**	23.56	22.77	**18.59**	**15.24**	NT
9	**14.13**	24.09	**19.71**	**16.63**	20.82	**18.78**	20.29	**18.00**	NT
10	**14.21**	**18.11**	**17.93**	**16.07**	27.80	NT	22.29	**18.32**	NT

NT–Not tested; ND–Not detected

**Table 5:** qRT-PCR results for viral transport medium containing swabs collected from mucosal surfaces of affected *M*. *georgesi* turtles using an assay detecting a segment of the putative replicase 1a gene. Results for samples with very high RNA loads (Ct<20) shown in bold. These animals correspond to the st 5 animals from which tissues were collected (Table 4).

Animal	Cloaca	Conjunctiva	Mouth
6	20.41	23.56	26.22
7	20.80	26.23	28.57
8	**19.53**	27.39	24.37
9	24.26	27.64	25.36
10	21.98	28.34	24.16

**Table 6:** qRT-PCR results for serum samples collected from affected *M*. *georgesi* turtles using an assay detecting a segment of the putative replicase 1a gene. Results for samples with very high RNA loads (Ct<20) shown in bold. Each is a different animal to those shown in Tables 4 and 5.

Animal	Serum	Animal	Serum
11	35.34	17	21.22
12	**19.38**	18	22.29
13	32.06	19	27.00
14	29.98	20	35.63
15	25.98	21	28.56
16	26.04		

### *In situ* hybridisation

**Fig 6:**
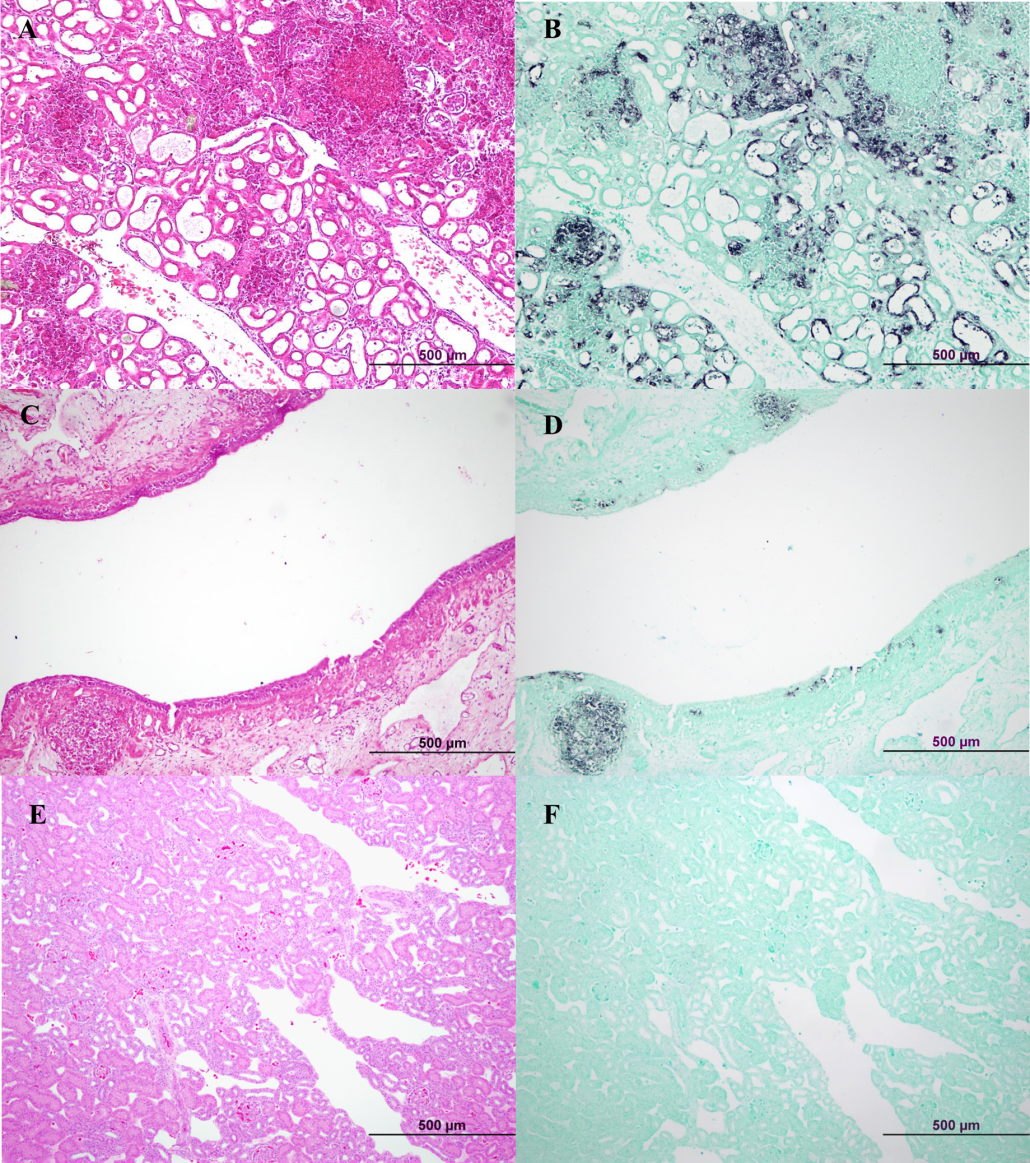
ISH detection of viral nucleic acid from Bellinger River virus associated with histopathological lesions in tissues of *M*. *georgesi*. (A) Haematoxylin and eosin stained kidney section with severe multifocal necrotizing nephritis. (B) Serial section of (A) with positive, dark purple ISH staining in areas of necrotizing inflammation as well as within degenerate or necrotic renal tubule cells, with Fast Green counterstain. (C) Haematoxylin and eosin stained bladder section with dense foci of necrotizing cystitis. (D) Serial section of (C) with dark purple ISH staining within areas of necrotizing cystitis as well as in scattered granulocytes in the urothelium, with Fast Green counterstain. (E) Haematoxylin and eosin stained kidney section from a BRV PCR negative Eastern long neck turtle (*Chelodina longicollis*). (F) Serial section of (E) is negative for ISH staining when treated with 5ng/μL of ISH probe, with Fast Green counterstain.

ISH was conducted on a selection of affected tissues and confirmed the presence of viral nucleic acid in association with severely necrotic histological lesions. Within a severely affected lacrimal gland there was staining of residual glandular epithelial cells and staining within areas of necrotising inflammation. The kidney lesions from two affected turtles showed similar staining in areas containing intense inflammatory infiltrates and necrotic cellular debris, within degenerate or necrotic renal tubule epithelial cells (Fig 6A and 6B) and within foci of vasculitis. Staining was also detected within dense foci of necrotising cystitis, as well as in scattered granulocytes in the multifocally oedematous urothelium (Fig 6C and 6D). Occasional granulocytes stained within the myocardial interstitium. No staining was observed in the negative control preparations which included tissue sections processed without the probe and tissues from an Eastern long neck turtle (*Chelodina longicollis*) that had given negative results by PCR).

### Survey results

Samples of all animals collected (Table 2) were tested in the replicase 1a qRT-PCR assay. Of the *Myuchelys georgesi* turtles tested (n = 31), viral RNA was detected in conjunctival swabs from eight animals and also in oral swabs from 2 of these animals. Ct values were consistently high (Ct >31), with most Ct >35. Low levels of viral RNA were also detected in conjunctival swabs from two of the 49 *Emydura macquarii* turtles tested and in 2 of 13 samples of egg casings of unknown species (Ct >37 in each instance) (Table 2). These casings had been adherent to the plastron of 2 *M*. *georgesi*. The reactivity detected in each of these samples collected during this field survey was confirmed with similar results obtained when tested in the replicase 1a qRT-PCR.

## Discussion

The virus described in this study, for which the name “Bellinger River virus” (BRV) is proposed, is believed to have had a profound impact on the wild *Myuchelys georgesi* population. The data presented provide strong, indirect evidence that this virus is the principal aetiological agent involved in the deaths of these *M*. *georgesi*. Unfortunately, as this is a now a critically endangered species of turtle [30], it is not possible to undertake experimental transmission studies to fulfil Koch’s postulates. Nevertheless, the criteria for disease causation defined by Fredericks and Relman [31] have been met. BRV, as a novel nidovirus, was isolated from tissues of diseased animals, very high levels of viral RNA were detected in tissues with marked pathological changes and *in situ* hybridisation assays demonstrated the presence of specific viral RNA in lesions in kidneys and eye tissue–two of the main affected organs. No or very low levels of viral RNA were detected in normal animals tested at the time of the outbreak. Collectively these data suggest that this virus is the likely cause of these mortalities. The high levels of viral RNA in several different organ systems would suggest that this virus is actively replicating in these organs and these detections are not an incidental finding or due to contamination as a result of either ingestion or inhalation of virus from the environment. Nevertheless, although we believe that BRV is the principal pathogen, it is inevitable that other factors are likely to have contributed to the onset and severity of disease. The turtles were probably already in a stressed and potentially immunosuppressed state as they had lost considerable body condition [2]. The higher water temperatures may have supported and perhaps enhanced virus replication, a phenomenon that is well known for a number of aquatic viruses [32, 33] and, although other pathogens have not been identified, it is likely that other microbes, even commensals, may have contributed to disease severity and ultimately the death of these turtles.

The *M*. *georgesi* population has been reduced to such an extent that the species has now been classified as “critically endangered” [30], with perhaps less than 100–200 animals present in the wild. The survival of the species may be dependent on a captive breeding population [2] due to the very small number of mature adults (possibly 10–15) that have survived in the wild (Chessman and Jones, Pers Comm [S1 File]. The impact of Bellinger River virus, the constrained geographical location of *M*. *georgesi*, the potential for hybridisation with *Emydura macquarii* and perhaps a range of environmental factors [2] have combined to seriously threaten the survival of this species of turtle. There are few documented instances where a pathogen has been directly incriminated in the potential extinction of an animal species [2] and to have such an impact in a very short time period. For example, while the chytrid fungus of *Batrachochytrium dendrobatidis* has resulted in significant declines in amphibian populations in a number of countries and on different continents [34, 35], its impact on amphibian populations has taken place over almost two decades, admittedly on a broad geographical scale.

At the time of its identification, this nidovirus was the first of the members of the proposed *barnivirus* genus (family *Coronaviridae*, subfamily *Torovirinae*) to be associated with disease in an aquatic reptile. Other *barniviruses* have been linked to infection and often disease in terrestrial reptiles. These include infections in ball pythons (*Python regius)* [18, 19, 36], Indian rock python, (*Python molurus)* [20, 36], Burmese python (*Python bivittatus*) [36], green tree python (*Morelia viridis*) [21], carpet python (*Morelia spilota*) [36], boa constrictor (*Boa constrictor*) [36] and shingleback lizards (*Tiliqua rugose*) [22]. In the terrestrial reptiles, the predominant clinical presentation has been respiratory disease [18–22, 37]. Experimental infections undertaken in ball pythons [37] have also demonstrated a tropism for the respiratory tract. In this respect, BRV differs in that, while there are pathological changes in several organ systems, the most severe changes are in the kidneys, corresponding to the high viral loads detected. It is interesting to note that there was only evidence of BRV replication *in vitro* in kidney cell cultures. While there was evidence of visible cytopathology in primate kidney cell cultures, virus replication was also detected in MDBK cells when culture supernatants were tested by qRT-PCR. The tropism of this virus for kidney cells would suggest that renal disease was a key factor contributing to the death of these turtles.

Chelonian species have been reported to be infected by a wide range of viral taxa including adenoviruses, bunyaviruses, flaviviruses, herpesviruses, paramyxoviruses, picornaviruses, ranaviruses, retroviruses and togaviruses [38, 39]. A virus in the order *Nidovirales*, family *Arteriviridae*, has been identified in the Chinese softshell turtle, *Pelodiscus sinensis*, during an outbreak of severe haemorrhagic disease affecting multiple organs including gonads, intestine, kidney, liver, lung, and spleen [40] and, recently, partial sequence of another virus from the *Arteriviridae* was detected by nucleic acid sequencing of the gut, liver and lungs of an apparently normal Chinese broad headed pond turtle (*Mauremys megalocephala*) [24].

Based on the extent of the epizootic in the Bellinger River, it would appear that BRV was introduced into a naive population. The origin of BRV is yet to be determined as there was no evidence of virus in other species sampled from the affected area, except for the detections of low levels of BRV RNA in *Emydura macquarii*, a closely related species that can interbreed with *M*. *georgesi* and in 2 clusters of egg casings. In each instance the viral RNA levels were close to the limit of detection and are of uncertain significance. The possibility that these are the result of superficial contamination from the environment cannot be excluded, and is likely for the egg casings as they were removed from the carapace of *M*. *georgesi* turtles in the Bellinger River.

BRV is the first nidovirus in the proposed *barnivirus* genus that has been isolated from a non-squamatid reptile and is phylogenetically placed between the recognised python nidoviruses and the shingleback lizard nidovirus. This may indicate that the *barniviruses* are quite widespread among both aquatic and terrestrial reptile species. The apparent abundance of reptilian nidoviruses would suggest that this turtle virus may have originated in a snake, lizard or other reptile and that *M*. *georgesi* could be an incidental host. However, as detections of viruses from the order *Nidovirales* have also been reported in fish [41–45] and more distantly related viruses even in insects [46–48], there are many potential reservoirs for this virus.

Finally, from a practical perspective, the detection of viral RNA in cloacal and ocular swabs, serum and plasma indicates that these are samples that could be collected from live endangered animals for future screening or surveillance. These sample types have already been used to select a group of presumptively virus-free animals to establish a captive breeding colony [2] that remains healthy and apparently free of virus after 2 years in isolation. Serum, conjunctival and cloacal swabs from the animals in this captive colony have given negative results in the BRV qRT-PCR assays on several occasions over this 2 year period. However, the development of serological assays to detect antibodies to this virus in various animal species would also be advantageous to provide additional confidence in the presumptive virus-free status of captive breeding colonies. These assays would also be invaluable to support epidemiological studies and to assist the search for the origins of this novel virus.

## Supporting information

S1 Fig Phylogenetic tree showing the genetic relationships of members of the sub-family *Torovirinae* based on the helicase region of polyprotein 1ab. (TIF)

S2 Fig Phylogenetic tree showing the genetic relationships of members of the sub-family *Torovirinae* based on the M^pro^ coding region of polyprotein 1a. (TIF)

S3 Fig Phylogenetic tree showing the genetic relationships of members of the sub-family *Torovirinae* based on the full length polyprotein 1a. (TIF)

S4 Fig Phylogenetic tree showing the genetic relationships of members of the sub-family *Torovirinae* based on the spike protein. (TIF)

S1 Table List of primers used for PCR amplification prior to undertaking Sanger sequencing to complete the genome. (DOCX)

S2 Table Alignment of the 268 amino acid residues within the conserved RNA-dependent RNA polymerase (RdRp) region of polyprotein 1ab of members of the sub-family *Torovirinae*, as used to construct the phylogenetic tree. (XLS)

S1 File Letter from B. Chessman and H. Jones describing estimates of the current size and composition of the *M*. *georgesi* population. (PDF)
