# Supplementary figures and images for: Identification of a novel nidovirus as a potential cause of large scale mortalities in the endangered Bellinger River snapping turtle (Myuchelys georgesi)
Source: PLoS One. 2018 Oct 24;13(10):e0205209. doi: 10.1371/journal.pone.0205209 (PMC6200216; doi:10.1371/journal.pone.0205209)

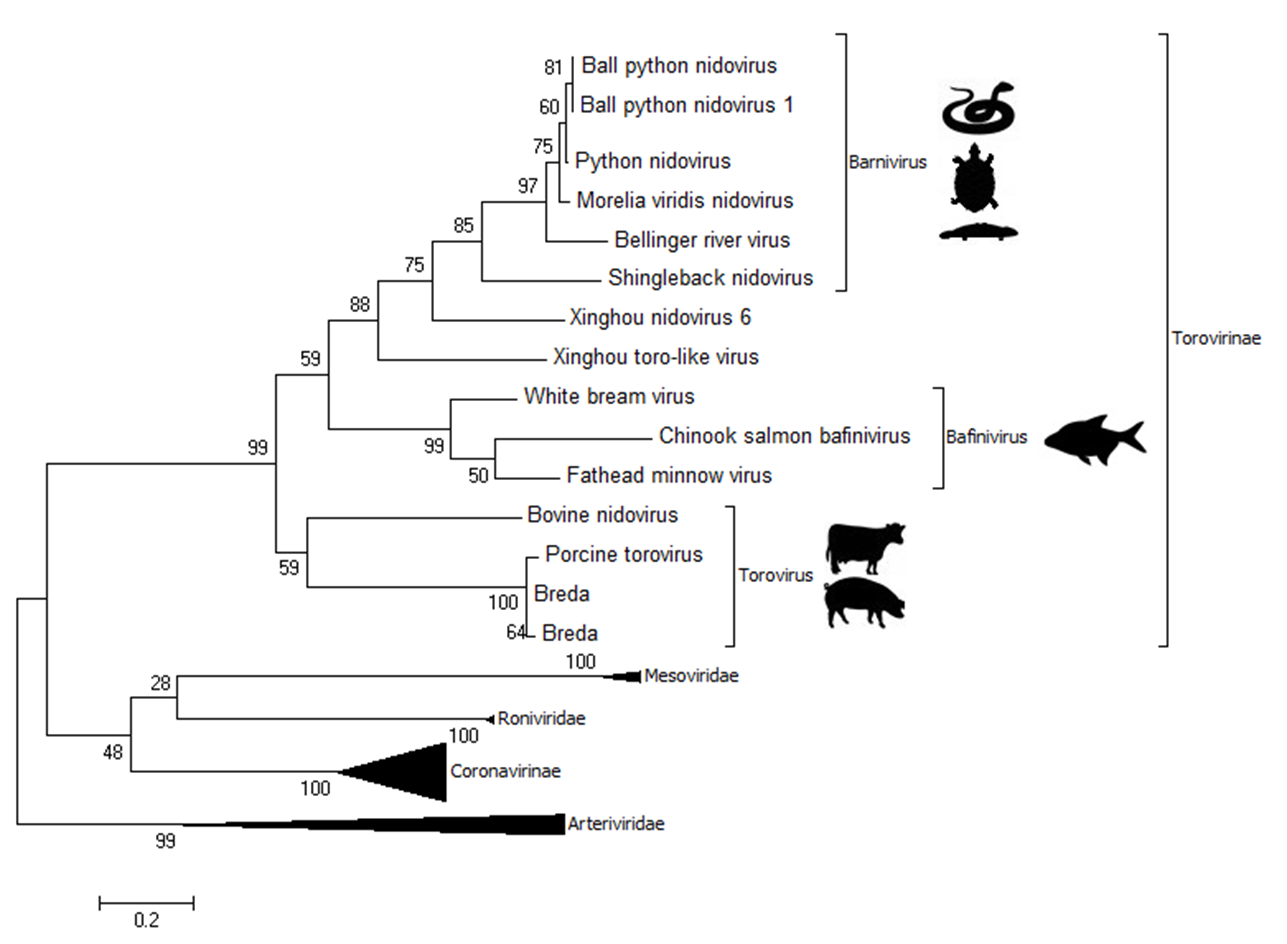

Supplement: S1 Fig — (TIF) [file pone.0205209.s001.tif]

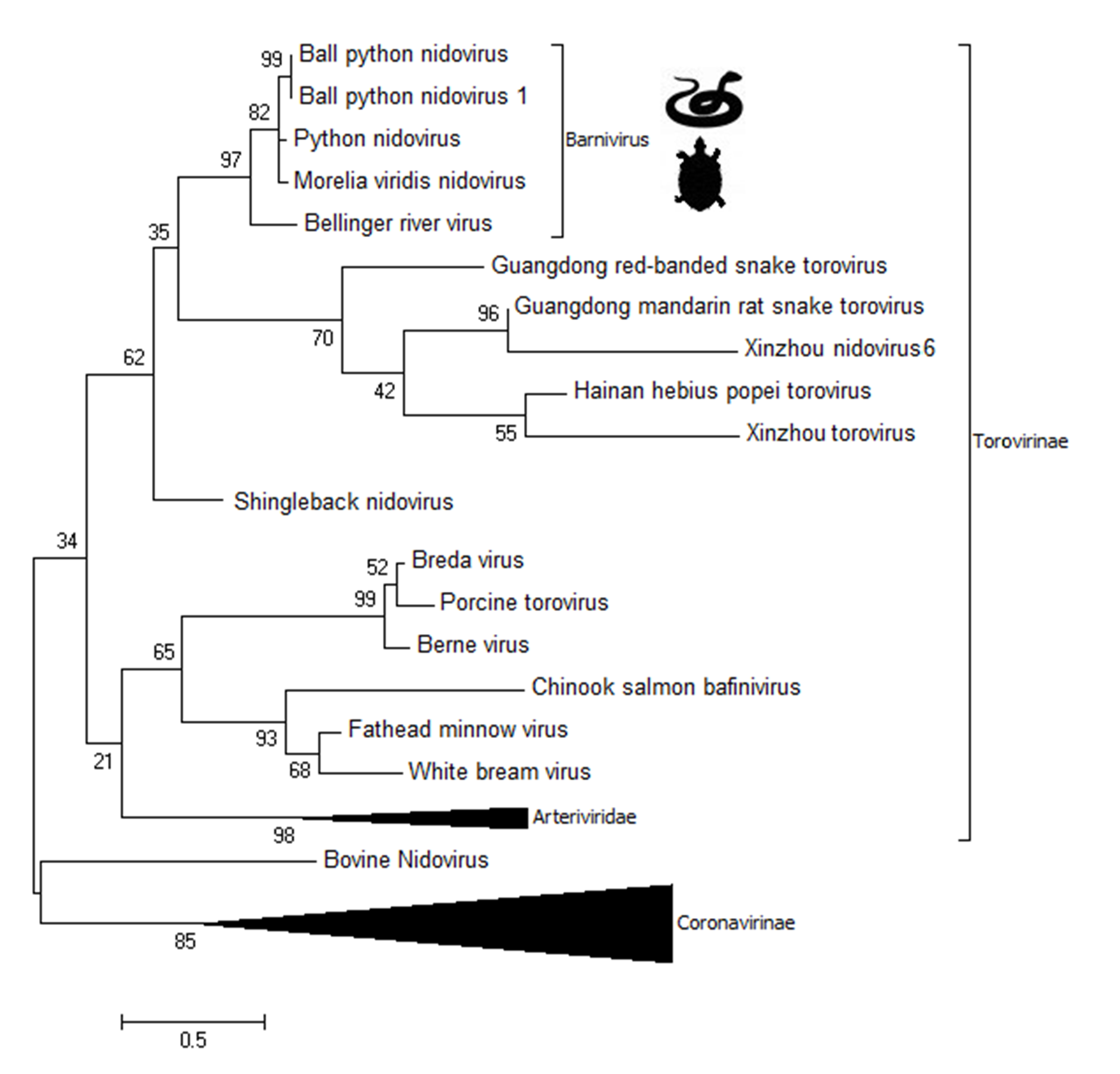

Supplement: S2 Fig — (TIF) [file pone.0205209.s002.tif]

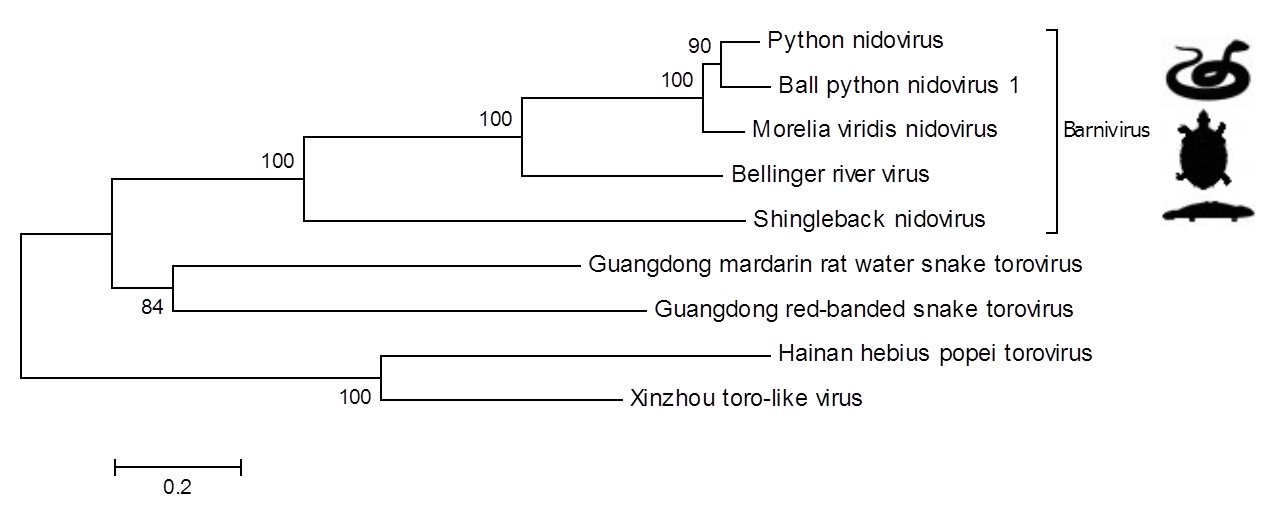

Supplement: S3 Fig — (TIF) [file pone.0205209.s003.tif]

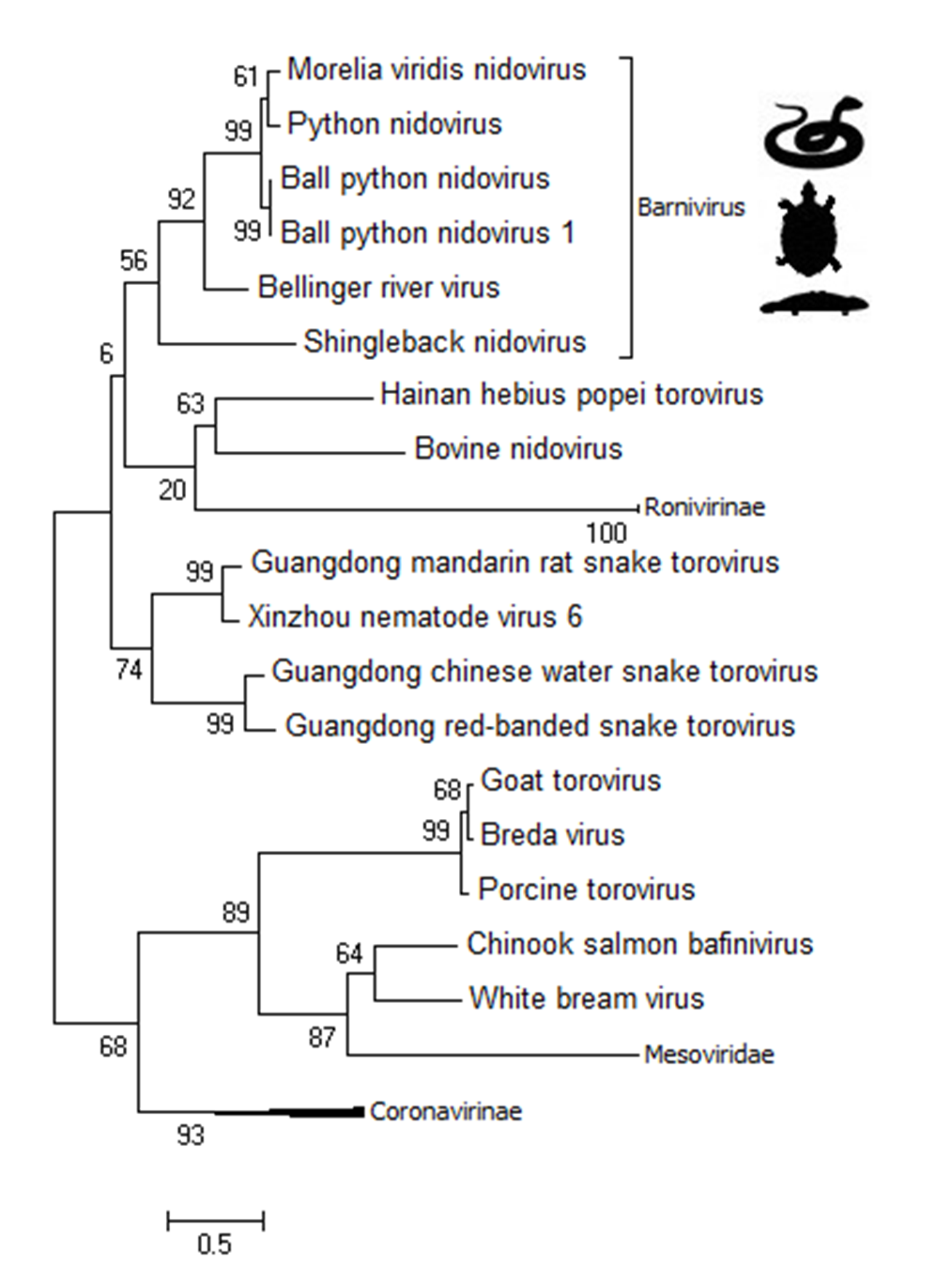

Supplement: S4 Fig — (TIF) [file pone.0205209.s004.tif]
