# Supplementary material for: Identification of a novel nidovirus as a potential cause of large scale mortalities in the endangered Bellinger River snapping turtle (Myuchelys georgesi)
Source: PLoS One. 2018 Oct 24;13(10):e0205209. doi: 10.1371/journal.pone.0205209 (PMC6200216; doi:10.1371/journal.pone.0205209)
Supplement: S1 Table — (DOCX) [file pone.0205209.s005.docx]

**Supplementary Table 1**

| Primer | Sequence 5’ to 3’ |
| --- | --- |
| TNV_F1_37 | AGCATTCTGAACCCCATTTGTTC |
| TNV_R5_6921 | GCGGTGATTGTAGATTGCAGG |
| TNV_F6_6927 | GAAGGCGACCACTACGGAA |
| TNV_R6_8270 | CGTTGTGTCCGTTGCCTTT |
| TNV_F7_8183 | GCCGACATCGCGCAAAAG |
| TNV_R7_9674 | TGATGAGACATGAGCCGCAG |
| TNV_F8_9563 | CCTATCACGATCCACTCCTGC |
| TNV_R8_11034 | TATGGTCTGGCGTTCGTGTT |
| TNV_F9_10948 | CGCACTTTGCATTCTACAGACC |
| TNV_R9_12439 | GCGTTCGGTGACTGTGTTGA |
| TNV_F10_12323 | CACGACACAGTCACCGCAA |
| TNV_R1_1532 | ATGGCTTGCTTGTAGCGTTG |
| TNV_R10_13812 | AGCGTGTCGTGGAATTTTGG |
| TNV_F11_13694 | CACAACACACTCATCGGCATC |
| TNV_R11_15021 | TCTGCGCTGTTCTTGGACTG |
| TNV_F12_14922 | CACTTTCCCAACGTCAACGC |
| TNV_R12_16410 | GTTGTTCAGTGCTTCGGCTG |
| TNV_F13_16266 | CGACTTCGACACAGTTAGCC |
| TNV_R13_17785 | AGTCGTTGATGTTGCCGTGT |
| TNV_F14_17642 | GCGCACTCCACAAACCAGTC |
| TNV_R14_19105 | CCCAGGCGAAGAAGTTGTCG |
| TNV_F15_18928 | GGCAACTTCACAATCCGCTG |
| TNV_F2_1402 | CTCGGGCGTCATAGTCAAGT |
| TNV_R15_20524 | GCGGTTGTAGTGTGGTCGTA |
| TNV_F16_20421 | TCCGAGACGAAAACCGCAAG |
| TNV_R16_21920 | TGCCGCCGATTTTGAGTGA |
| TNV_F17_21765 | TCGACAACAACACCGAAGCAG |
| TNV_R17_23352 | GTGAGTTGCAGGCTGTTGTCT |
| TNV_F18_23262 | CAACTCACGCAAGCACTACC |
| TNV_R18_24744 | GCGAGCGGTCTGGAAAGAA |
| TNV_F19_24612 | GTCGCTAAAGTCCAAGCCC |
| TNV_R19_26141 | TGCTGGTTCGTCTTAGTCTTGT |
| TNV_F20_26058 | ACACCAACTCATGCCTCCAC |
| TNV_R2_2837 | CCTCGATGGCGTTGGTAAGT |
| TNV_R20_27549 | TTGGTTCCAGGTCGTTTGGG |
| TNV_F21_27444 | TCGTACCACGAGACCTCAGC |
| TNV_R21_28965 | TGAGGAGTGTGAGTTGGCATT |
| TNV_F22_28836 | GGCGCATCAGGTACAGCATTA |
| TNV_R22_30296 | CGTGCTCACTTCAAAGGTGTT |
| TNV_F23_29740 | CGTCGAGTGTCTAACAGGCA |
| TNV_R23_30480 | TTCTAGCTGACGGAGCGGT |
| TNV_F3_2734 | GAACATCGGCAAAACCTCACT |
| TNV_R3_4184 | TCTTTTCGGCTTTGGCTTGTG |
| TNV_F4_4081 | TAACACCGAAAGCAGCACAC |
| TNV_R4_5568 | TGGTTGCTTGTAGCTCTCCG |
| TNV_F5_5431 | CATCCGCTACCACCTACGC |
| TNV_F1_37 | AGCATTCTGAACCCCATTTGTTC |
| TNV_R5_6921 | GCGGTGATTGTAGATTGCAGG |
| TNV_F6_6927 | GAAGGCGACCACTACGGAA |
| TNV_R6_8270 | CGTTGTGTCCGTTGCCTTT |
| TNV_F7_8183 | GCCGACATCGCGCAAAAG |
| TNV_R7_9674 | TGATGAGACATGAGCCGCAG |
| TNV_F8_9563 | CCTATCACGATCCACTCCTGC |
| TNV_R8_11034 | TATGGTCTGGCGTTCGTGTT |
| TNV_F9_10948 | CGCACTTTGCATTCTACAGACC |
| TNV_R9_12439 | GCGTTCGGTGACTGTGTTGA |
| TNV_F10_12323 | CACGACACAGTCACCGCAA |
| TNV_R1_1532 | ATGGCTTGCTTGTAGCGTTG |
| TNV_R10_13812 | AGCGTGTCGTGGAATTTTGG |
| TNV_F11_13694 | CACAACACACTCATCGGCATC |
| TNV_R11_15021 | TCTGCGCTGTTCTTGGACTG |
| TNV_F12_14922 | CACTTTCCCAACGTCAACGC |
| TNV_R12_16410 | GTTGTTCAGTGCTTCGGCTG |
| TNV_F13_16266 | CGACTTCGACACAGTTAGCC |
| TNV_R13_17785 | AGTCGTTGATGTTGCCGTGT |
| TNV_F14_17642 | GCGCACTCCACAAACCAGTC |
| TNV_R14_19105 | CCCAGGCGAAGAAGTTGTCG |
| TNV_F15_18928 | GGCAACTTCACAATCCGCTG |
| TNV_F2_1402 | CTCGGGCGTCATAGTCAAGT |
| TNV_R15_20524 | GCGGTTGTAGTGTGGTCGTA |
| TNV_F16_20421 | TCCGAGACGAAAACCGCAAG |
| TNV_R16_21920 | TGCCGCCGATTTTGAGTGA |
| TNV_F17_21765 | TCGACAACAACACCGAAGCAG |
| TNV_R17_23352 | GTGAGTTGCAGGCTGTTGTCT |
| TNV_F18_23262 | CAACTCACGCAAGCACTACC |
| TNV_R18_24744 | GCGAGCGGTCTGGAAAGAA |
| TNV_F19_24612 | GTCGCTAAAGTCCAAGCCC |
| TNV_R19_26141 | TGCTGGTTCGTCTTAGTCTTGT |
| TNV_F20_26058 | ACACCAACTCATGCCTCCAC |
| TNV_R2_2837 | CCTCGATGGCGTTGGTAAGT |
| TNV_R20_27549 | TTGGTTCCAGGTCGTTTGGG |
| TNV_F21_27444 | TCGTACCACGAGACCTCAGC |
| TNV_R21_28965 | TGAGGAGTGTGAGTTGGCATT |
| TNV_F22_28836 | GGCGCATCAGGTACAGCATTA |
| TNV_R22_30296 | CGTGCTCACTTCAAAGGTGTT |
| TNV_F23_29740 | CGTCGAGTGTCTAACAGGCA |
| TNV_R23_30480 | TTCTAGCTGACGGAGCGGT |
| TNV_F3_2734 | GAACATCGGCAAAACCTCACT |
| TNV_R3_4184 | TCTTTTCGGCTTTGGCTTGTG |
| TNV_F4_4081 | TAACACCGAAAGCAGCACAC |
| TNV_R4_5568 | TGGTTGCTTGTAGCTCTCCG |
| TNV_F5_5431 | CATCCGCTACCACCTACGC |
| TNV_SF_822 | GGTCTTCTTAGTACCACTTCG |
| TNV_SF_6655 | TCATCGAAGGACTCATCAAC |
| TNV_SR_6038 | AGTTGGACAGTGTATCTTGG |
| TNV_SF_7481 | GCTGCAAACTCAAGAAGAAC |
| TNV_SR_7745 | AGTGTCGACTGTACTTGAAC |
| TNV_SF_7910 | GACAACTCCGAGTATACCAC |
| TNV_SR_8490 | GAGATTACTACGGCAGAAGG |
| TNV_SR_8920 | CCTTCTGCCGTAGTAATCTC |
| TNV_SF_9395 | CAACTGACCAACATCAACAC |
| TNV_SF_10018 | TACTCTACATGTCACACGAC |
| TNV_SF_10642 | ATCACAACGCTACTCATCAG |
| TNV_SR_838 | ATTACGGGTCTTACGAAGTG |
| TNV_SR_10399 | ATGTGTCGGTTGCTATAGTC |
| TNV_SR_11550 | CGTAGTACTGGATGCTGTTG |
| TNV_SR_12095 | TCTTGATGTAGTCTTGGTCG |
| TNV_SF_12145 | AAGTTACCACACGAGTTCAG |
| TNV_SF_13438 | ATCAACAAAGCACAACTCAC |
| TNV_SF_13075 | TACAGGATGAACAGGATGAC |
| TNV_SR_16264 | CTGTCTGATTGCTTTGAGTC |
| TNV_SF_15529 | GAAACCAAATCACATGGGAC |
| TNV_SR_16315 | GAAATCCTCAGAGACCTGC |
| TNV_SR_15676 | TGTAGTTTGCCGTACAGTG |
| TNV_SR_1408 | TACCGAACTTGACTATGACG |
| TNV_SR_16949 | CAGTGAGAGACCAGCAAC |
| TNV_SF16136 | CCAAATACCACAACACGAAG |
| TNV_SF_17451 | AGGTAAGCTCTTCATCCAAC |
| TNV_SR_18491 | TGCTTGTTGTAGATGTCTGG |
| TNV_SF_18696 | CACCAGACATGACACTCATC |
| TNV_SF_20240 | TTCGAACCAGCTAAAGAAGG |
| TNV_SR_20020 | TCGTGAACGGATGATTGC |
| TNV_SF_21720 | ATTCTCATCGACTGGCAAG |
| TNV_SR_21420 | ATCTGGTTGATGATCGTGC |
| TNV_SF_22736 | GACGAACACAACCAAATACC |
| TNV_SR_1768 | TAGAATGCCTTTGAGTCCTG |
| TNV_SF_23223 | GACACAATCCTACGAGCC |
| TNV_SR_22781 | GAACTGAGCCTTGTTTGAAG |
| TNV_SR_24312 | CGCAGAATGTCTTGAAGATG |
| TNV_SF_24585 | AGAATCGAAGACTGCACAG |
| TNV_SF_25556 | AGAAGCTACTCATCGTCAAC |
| TNV_SR_25941 | GAGCATGTCTCCTAGTGATG |
| TNV_SR_25211 | CGACTTGTTGGAAGTTCTTG |
| TNV_SF_27219 | CCGAAGACTACATCTACCAC |
| TNV_SR_27488 | GTTTGGATCTGGTTTGACTG |
| TNV_SR_26820 | TCTTTCGGATGATGAACCAG |
| TNV_SF_2508 | CTCAAACCATCCGAAACATG |
| TNV_SF_28752 | AGCATCCAAAGTCCTACATG |
| TNV_SR_28330 | CGTGGTTGAAATACTGGATG |
| TNV_SF_29494 | GGCAGTAACAAATTCGAGAG |
| TNV_SR_29887 | AGCGGTATTTCTTGTGTTTG |
| TNV_SR_12906 | CTGTCTGATTGCTTTGAGTC |
| TNV_SF_3857 | ACACTATCAAGTTCACCGAC |
| TNV_SR_3755 | TTTGTGGATATAGTGGCAGG |
| TNV_SF_5135 | ACCAATTCCTAGCCGAATAC |
| TNV_SR_4752 | CGTTGATGAATGTGTTGAGG |
| BRVF_686 | GGCATAGTCAGCCTAATTGT |
| BRVF_7470 | CAGTCAACATGCTAACTCAAG |
| BRVF_13066 | GATGATATACAGGATGAACAGGA |
| BRVF_14782 | CATCTTATCACTGCGACTCA |
| BRVR_22815 | GTTCACGTACTTGATGAGGA |
| BRVF_23746 | CTACCACAACTGTACGAGC |
| BRVF_22575 | CCGCAGACTAAAGTCTTACA |
| BRVR_23927 | GTCGATCTGGCTGTTGATAA |
| BRV1075SR | ATAGTTGGCCACTGAGGTA |
| BRV4130SR | TGTCGGATTGCGGTTTC |
| BRV4463SF | AACATCGAAAGGACCACATC |
| BRV8357SR | CCGTTGCCTTTGTAGATGAG |
| BRV9041SR | ATGTGTTGAGTGCTGTGG |
| BRV10239SR | GTCGGTGCTGTCGTAATAG |
| BRV14215SR | GAGCACTTGGTGACTTGTT |
| BRV16230SR | GCTTTGAGTCGGTCTTTGA |
| BRV16510SR | CAGTTCGTTGTTCAGTGCTTC |
| BRV17388SR | AGGACGCTGTCGATGTTTC |
| BRV17115SF | GTCACCGGAACACACAAA |
| BRV19849SF | AGTGAGCAGTGAGCATTC |
| BRV25899SR | CGCAGACTAGGAACCATTTG |
| BRV25310SF | TCGCAAGTGAACATCAAATC |
| BRV25945SF | CTCGGCATCTCAGTCATTAAA |
| BRV25219SR | GATTTCGCTCATTCGGTAGTC |
| BRV26763SF | CATCACCGACTCAACATCAC |
| BRV27421SR | CTTGTTCTGGTGTGATGAGTAG |
| BRV29337SR | GTCTGATGGAGTTGTGACTG |
| BRV20475F | TCTTACCAGGCCTAATG |
| BRV21207R | GTTGGTTGATGGTTTGA |
| BRV21949F | GAGCCATCACTCTTCAG |
| BRV22865R | GTTCGATCGTTTGGTTG |
| BRV22638F | CGTCTTAGACAACTTCTTC |
| BRV23580R | GTTGCTGATTGAGCTTG |
| BRV24005F | CACTAAGTCGATCCTACG |
| BRV24945R | ATGTCTGCTTTGCTGTT |
