# Supplementary material for: Identification of a novel nidovirus as a potential cause of large scale mortalities in the endangered Bellinger River snapping turtle (Myuchelys georgesi)
Source: PLoS One. 2018 Oct 24;13(10):e0205209. doi: 10.1371/journal.pone.0205209 (PMC6200216; doi:10.1371/journal.pone.0205209)
Supplement: S1 File — (PDF) [file pone.0205209.s007.pdf]

Dr Peter Kirkland
Senior Principal Research Scientist
Officer in Charge, Virology Laboratory
Elizabeth Macarthur Agricultural Institute
Woodbridge Road, Menangle NSW 2568
Australia

Dear Dr Kirkland,

Population estimate for the Bellinger River turtle

Thank you for your enquiry about the size of the remaining wild population of the Bellinger River turtle, *Myuchelys georgesi*.

Using data from a snorkelling survey of the Bellinger River conducted by the New South Wales Office of Environment and Heritage in November 2017, and parametric statistical methods described by Thompson S.K. (2012), Sampling (3rd edition), John Wiley & Sons, I have estimated the total population of *M. georgesi* in the river at that time as 109 individuals. Only about 15% of the population are adults or subadults.

My colleague Dr Hugh Jones has produced an alternative estimate of turtle detectability by a nonparametric bootstrapping method, which yields a population estimate of 127 individuals.

These estimates have considerable uncertainty because of factors such as sampling variability, uncertainty about the total area of *M. georgesi* habitat, and the possibility that some turtles are able to avoid detection by snorkelers through cryptic behaviour. However, I consider it unlikely that the population exceeds 200 individuals.

Dr Jones and I intend to include population estimates for *M. georgesi* in a manuscript for submission to a peer-reviewed scientific journal, which is currently in preparation.

Yours sincerely,

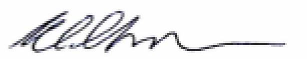

Dr Bruce Chessman
Chessman Ecology
7 Dalrymple Crescent
Pymble NSW 2073
Australia

20 August 2018
